# Let’s Talk about Placental Sex, Baby: Understanding Mechanisms That Drive Female- and Male-Specific Fetal Growth and Developmental Outcomes

**DOI:** 10.3390/ijms22126386

**Published:** 2021-06-15

**Authors:** Ashley S. Meakin, James S. M. Cuffe, Jack R. T. Darby, Janna L. Morrison, Vicki L. Clifton

**Affiliations:** 1 Early Origins of Adult Health Research Group, UniSA: Clinical and Health Sciences, University of South Australia, Adelaide, SA 5000, Australia; ashley.meakin@unisa.edu.au (A.S.M.); Jack.Darby@unisa.edu.au (J.R.T.D.); janna.morrison@unisa.edu.au (J.L.M.); 2 School of Biomedical Sciences, The University of Queensland, Brisbane, QLD 4072, Australia; j.cuffe1@uq.edu.au; 3 Mater Medical Research Institute, The University of Queensland, Brisbane, QLD 4000, Australia

**Keywords:** placenta, fetal growth, sex differences, androgens, glucocorticoids, androgen receptor, glucocorticoid receptor, steroid hormone signaling, pregnancy, fetal development

## Abstract

It is well understood that sex differences exist between females and males even before they are born. These sex-dependent differences may contribute to altered growth and developmental outcomes for the fetus. Based on our initial observations in the human placenta, we hypothesised that the male prioritises growth pathways in order to maximise growth through to adulthood, thereby ensuring the greatest chance of reproductive success. However, this male-specific “evolutionary advantage” likely contributes to males being less adaptable to shifts in the in-utero environment, which then places them at a greater risk for intrauterine morbidities or mortality. Comparatively, females are more adaptable to changes in the in-utero environment at the cost of growth, which may reduce their risk of poor perinatal outcomes. The mechanisms that drive these sex-specific adaptations to a change in the in-utero environment remain unclear, but an increasing body of evidence within the field of developmental biology would suggest that alterations to placental function, as well as the feto-placental hormonal milieu, is an important contributing factor. Herein, we have addressed the current knowledge regarding sex-specific intrauterine growth differences and have examined how certain pregnancy complications may alter these female- and male-specific adaptations.

## 1. Introduction

Evidence of sex-specific intrauterine growth rates and outcomes have been observed throughout gestation with males, on average, having accelerated growth rates and increased growth outcomes relative to females [1,2,3,4,5,6,7,8,9,10,11,12,13]. In fact, the first observation of sex-specific neonatal outcomes was reported by Dr. Clarke during the late 18th century [14]. Dr. Clarke’s *Observation on the Mortality of Male Children* reported from more than 20,000 cases that males on average had an increased birthweight when compared with females. Importantly, the work of Dr. Clarke also noted an increase in the rates of mortality of male fetuses and neonates, an observation that has since been consistently reported in several human pregnancy cohorts. It has been argued that males “live dangerously in the womb” [15] by prioritising feto-placental growth pathways at the cost of placental reserve capacity when compared with females. Indeed, our group and others have reported sex-specific rates in small for gestational age (SGA), preterm delivery, and additional intrauterine and neonatal morbidities and mortality in the presence of pregnancy complications—including maternal asthma, preeclampsia (PE), and gestational diabetes mellitus (GDM)—whereby males are at a greater risk of adverse outcomes [16,17,18,19,20,21]. The mechanisms that drive these sex-specific risks remain largely unknown, but alterations to physiological processes that are both dependent and independent on placental function and structure are thought to be critical, determining factors.

## 2. From Blastocyst to Neonate: Ontogeny of Sex-Specific Intrauterine Growth

The apparent male-specific growth phenotype has been observed as early as the pre-implantation period in several mammalian species, including humans. In vitro pre-implantation studies have identified that XY bovine and human embryos have an overall increase in total cell mass, when compared with XX embryos. The mechanisms that contribute to these sex-specific differences in growth rates prior to implantation and establishment of the feto-placental unit may be, in part, the result of chromosome X-inactivation [3,22]. This female-specific alteration during early development may have implications for transcript expression and subsequent cellular function. Work conducted by Bermejo-Alvarez et al. [23] reported sex-specific patterns in the expression of mRNA transcripts involved in metabolic processes and cell cycle progression in the bovine pre-implantation embryo, such that there was an increase in males relative to females. These findings support previous work identifying an increase in total glucose metabolism in XY bovine pre-implantation embryos [24]. Despite the findings from several in vitro pre-implantation studies identifying sex-specific differences in transcript expression, growth rates, and total cell mass, a 2017 study examining the ontogeny of sex-specific growth and development reported no change in the total cell count of mouse pre-implantation embryos at E5 between the sexes [11]. These findings may be due to differences between in vitro and in vivo methodologies used but could also be the result of species-specific developmental differences.

**Figure 1:**
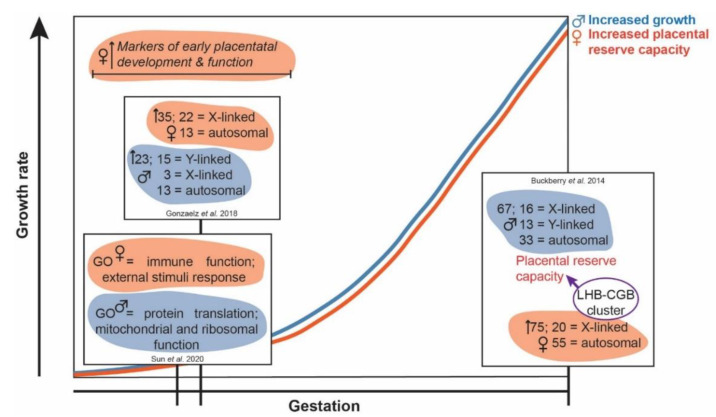
Alterations in female and male placental transcript expression across gestation may contribute to sex-specific feto-placental growth and function outcomes. It is postulated that the differential expression of transcripts in females increases placental reserve capacity and feto-placental adaptability to an altered maternal environment at the expense of a reduced growth trajectory, relative to males. GO = gene ontology; LHB-CGB = luteinising hormone beta subunit and chorionic gonadotropin beta subunit.

In humans, sex-specific differences in placental transcript expression during the early gestational period have been reported and may influence both fetal growth trajectories and outcomes. Using chorionic villi samples obtained for genetic testing at 10.5–13.5 weeks gestation, Gonzalez et al. [25] reported 58 transcripts were differentially expressed between the sexes, with 40 of these transcripts being chromosome X or Y-linked. Supporting previous work in the pre-implantation embryo, male first trimester placentae had enriched expression of transcripts involved in metabolic processes. More recent work by Sun et al. [26] reported sexual dimorphism in single-cell transcriptomic profiles of first trimester human placentae. In trophoblasts, gene ontology analysis of differentially expressed transcripts identified male trophoblast cells had enriched protein translation and certain mitochondrial and ribosomal functions, whereas female trophoblast cells had an enrichment in transcripts involved in immune function and responses to various compounds and stimuli. Overall, the pre-implantation male embryo and the early gestation male placentae preferentially increase transcripts involved in growth regulatory pathways, whereas age-matched females appear to prioritise alternative pathways. These sex-specific differences likely contribute to an observed increase in male growth rates and outcomes during early gestation in placental mammalian species including humans [1,27], but are also thought to contribute to the observed sex-specific differences in growth trajectories and outcomes during the second and third trimesters [12] (Figure 1).

Changes to placental structure and function between the sexes are less clearly reported in uncomplicated human pregnancies during the second and third trimesters, due to (i) the nature of early human placental delivery; or (ii) studies in late gestation human pregnancy cohorts focusing on birth outcomes. However, we can gain insight from pregnant animal models where some studies have shown sex-specific placental structural and functional differences across gestation. In an ontological study using rats, Kalisch-Smith et al. [11] reported an increase in total placental volume in male placentae at 15d gestation (term, 23d), and upregulated expression of genes related to vasculogenesis at 13d gestation in females. Similarly, work by O’Connell et al. [7] reported sexually dimorphic placental development throughout gestation in the spiny mouse. At 20–39d gestation (term, 39d), sex-specific differences in placental structure (as determined by the area of the labyrinth and junctional zones) and function (as determined by gene expression) were observed. Specifically, in males there was an increase in junctional zone area across gestation but a decrease in the labyrinth and labyrinth:junctional zone ratio, which is consistent with previous work by the same group [28]. The labyrinth zone functions as the site of nutrient and oxygen exchange; reduced labyrinth zone area has been implicated in reduced nutrient and oxygen delivery to the fetal circulation [29]. Likewise, increased junctional zone area has been reported in some studies to result in IUGR [30]. Thus, these sex-specific differences in placental architecture are thought to necessitate a greater functional reserve for the female placenta and likely contributes to reduced intrauterine rates of morbidities and mortality in the presence of an adverse maternal environment [6]. Functionally, male placentae had increased insulin-like growth factor (Igf) type 1 (*Igf1*), Igf1-receptor (*Igf1r*), and solute carrier (Slc) family 2 member 1 (*Slc2a1*) gene expression in both the labyrinth and junctional zone at 30–37d gestation, whereas female placentae had increased glucocorticoid receptor (*Nr3c1*) gene expression at 37d gestation in the labyrinth zone only. These sex-specific differences in gene expression may be an attempted male-specific adaptation to mitigate the growth-inhibitory effects of reduced labyrinth zone volume and labyrinth:junctional zone ratio [29]. In mice, male placentae at the end of gestation had increased expression of *Nr3c1*, vascular endothelial growth factor (Vegf) A (*Vegfa*), *Igf1r*, *Igf2r*, and *Slc38a1* expression, when compared to females, whereas female placentae had increased *Igf2* and *Slc2a1* expression [31]. This highlights that similar pathways are regulated by placental sex, even if the direction of change is different between species.

Although there is an apparent male-specific bias for upregulating growth signalling pathways in utero, current literature consistently demonstrates a greater change in feto-placental transcript expression in females relative to males across gestation. These sex-specific differences are observed in both uncomplicated pregnancies, as well as pregnancies complicated by distinct maternal pathologies. Microarray data of the mouse placenta from an uncomplicated pregnancy identified 232 differentially expressed autosomal transcripts between the sexes, with 199 of those being upregulated in female placentae relative to males [32]. The work by Mao et al. identified that of these 199 upregulated transcripts, eight encoded for olfactory receptors (*Olfr91*, *Olfr433*, *Olfr520*, *Olfr786*, *Olfr1381*, *Olfr1383*, *Olfr1394*, and *Olfr1395*). Although the physiological relevance of the olfaction system within the placenta remains poorly understood, the authors suggested that a female-specific bias for this system may contribute to increased adaptation to fetal and maternal changes in the levels of specific compounds. More recently, sex-specific differences of the transcriptomic signatures of female and male rat placentas (21d gestation) were identified. Unlike previous microarray analyses showing greater changes in females relative to males, the work of Lin et al. [33] identified 287 differentially expressed transcripts, with 159 being upregulated in male rat placentae. Subsequent KEGG pathway analysis identified genes involved in the cell adhesion molecules (CAMs) pathway to be enriched in male placentae. The physiological relevance of this finding was not discussed; however, work by others has identified an important role for CAMs in mediating appropriate placentation [34]. Therefore, the observed enrichment of the CAMs pathway in the late gestation male placentae may be an attempted male-specific compensatory adaptation to ensure continued placental invasiveness, perhaps to mitigate adverse events in late gestation brought on by suboptimal placentation.

In the first trimester of uncomplicated human pregnancies, the expression of specific placental biomarkers (soluble fms-like tyrosine kinase-1 (s-Flt1), placental growth factor (PlGF), and plasminogen activator inhibitor (PAI-2)) were significantly increased in the maternal serum of study participants carrying a female fetus [8]. These findings favour previous in vivo work that reported a more vascularised female placenta throughout gestation [7,11], and further indicate a female-specific bias for enhanced placental reserve capacity in the absence of any maternal complication. These findings by Brown et al. [8] are supported by a more recent microarray meta-analysis of term placentae from uncomplicated pregnancies: Buckberry et al. [35] identified 142 differentially expressed transcripts between the sexes, with 75 transcripts being upregulated in females. Pathway analysis of these sex-specifically expressed transcripts revealed an enrichment of canonical pathways involved in eukaryotic initiation factor 2 (EIF2), mammalian target of rapamycin (mTOR), and VEGF signalling. The study further identified a luteinising hormone beta subunit and chorionic gonadotropin beta subunit (LHB-CGB) cluster of seven genes (*LHB*, *CGB*, *CGB5*, *CGB7*, *CGB8*, *CGB1*, and *CGB2*) in female placentae. These genes are involved in the synthesis of luteinising hormone (LH) and human chorionic gonadotropin (hCG) hormone subunits. Both hormones are secreted by the placenta and bind to the same transmembrane receptor—luteinsing hormone/choriogonadotropin receptor (LHCGR)—that induces cyclic adenosine monophosphate (cAMP) expression [36], which may function to promote placental growth and angiogenesis [37,38]. Therefore, a female-specific bias for the LHB-CGB cluster may enhance placental growth and vasculogenesis and subsequently increase female placental reserve capacity when compared with males.

These changes in placental transcript expression are likely brought on by sex-specific differences in the placental methylome, with some studies reporting an increase in the methylation of CpG sites in male placentae, relative to females [39,40]. Braun et al. [41] identified that in female placentae, differentially expressed transcripts were enriched in extracellular matrix, mRNA splicing, and chromatin organisation, and subcellular associations included the extracellular matrix and the nucleus. To contrast this, and to support previous work, differentially expressed transcripts in male placentae were centered on oxidative phosphorylation and cellular catabolic processes, and subsequent gene ontology cellular compartment analysis reinforced associations with the mitochondrial compartment. Evidently, there are sex-specific differences in placental structure and function in several mammalian species. These changes consistently show females upregulate transcripts involved in pathways that may increase placental reserve capacity. In contrast, males appear to preferentially upregulate growth and metabolic regulatory pathways that may drive in utero growth; however, it has been postulated that this male-specific adaptation increases the risk of intrauterine morbidities and mortality in the presence of an adverse maternal environment [6,14,15,42]. Sex-specific alterations to placental transcript expression likely contribute to observed differences in placental function and structure between the sexes.

## 3. Sex-Specific Differences in Placental Function and Structure Contribute to Adverse Intrauterine and Neonatal Outcomes

Disrupted placental structure—often characterised by suboptimal placental vasculature—impairs delivery of nutrient-rich, oxygenated blood to the developing fetus. This, in turn, can be detrimental to appropriate fetal growth and development; some studies have shown altered placental structure and function to be more prevalent in males in the presence of specific pregnancy complications. For example, a recent study identified an increase in the uterine artery pulsatility index of study participants carrying a male fetus associated with a complicated pregnancy at 16–24 weeks gestation, indicative of reduced placental function [42]. These recent findings support the main findings of a 2020 meta-analysis of fetal sex and maternal pregnancy outcomes that reported an overall increase in cardiovascular and metabolic disease burden in the presence of a male fetus [21]. This apparent sexual dimorphism may manifest from suboptimal male implantation and placentation resulting in increased utero-placental resistance [42,43]. These studies further support the concept of reduced placental reserve capacity in males via changes in placental function and structure that likely contribute to increased risks of intrauterine morbidities (preterm delivery, SGA, intrauterine growth restriction (IUGR)) and mortality in the presence of an adverse maternal environment, when compared with females exposed to similar in utero conditions.

**Figure 2:**
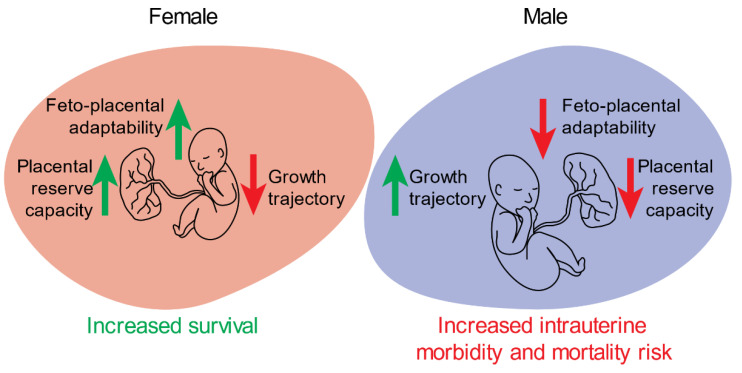
Overview of sex-specific in utero adaptations that drive differences in growth and survival outcomes. In females, increased feto-placental adaptability and placental reserve capacity result in increased survival rates at the expense of a reduced growth trajectory, whereas the opposite is observed in males.

Sex-specific differences in placental morphometry have been reported in human pregnancies complicated by maternal asthma, with the greatest reduction in absolute fetal capillary volume being observed in males [44]. Changes in angiogenic and vasculogenic factor expression in the placenta may be an important regulator of these observed outcomes. Several studies have reported sex-bias in the placental expression of these factors in both normal pregnancies and those complicated by maternal asthma or an SGA neonate [35,45,46], which may contribute to observed sex-specific differences in placental vasculature structure and function. Microarray data from a pregnancy cohort of maternal asthma reported no changes in the placental expression of *VEGFA*; however, *PlGF* expression was significantly decreased in female placentae from untreated asthmatic pregnancies, relative to non-asthmatic control, suggesting some level of appropriate placental response to a hypoxic maternal environment in females [45,46,47,48,49]. More recent work identified an increase in *VEGFA* mRNA expression in male placentae from asthmatic pregnancies, but no change in females [50]. This disparity in *VEGFA* expression between the sexes in the presence of maternal asthma further suggests an increased placental reserve capacity for female placentae and a reduced overall need for female-specific adaptations in the presence of severe maternal complications. However, in placentae from preeclamptic pregnancies, the expression of VEGF protein was significantly reduced in males and this was associated with an increase in hypoxia-inducible factor 1-alpha (HIF-1α) expression as well as an increase in apoptotic and pro-inflammatory markers when compared with females [51]. Evidently, sex-specific responses to the presence of a complication may be altered by certain pathophysiological conditions, but overall male placentae are less adaptable (Figure 2).

Although there is clearly no single, all-encompassing placental-mediated mechanism that drives these sex-specific intrauterine outcomes, but rather a combination of several pathways that may function (or dysfunction) synergistically to cause these outcomes, the literature has overlooked the effect of the feto-placental hormonal milieu for some time. Indeed, given that the placenta is recognsied as an endocrine organ, an increasing body of evidence has emerged to suggest that placental-specific, steroid-dependent regulation of female and male intrauterine growth outcomes exists, and that the dysfunction of the feto-placental hormonal milieu may contribute to adverse intrauterine outcomes between the sexes.

## 4. Changes to the Feto-Placental Hormonal Milieu Contribute to Sex-Specific Intrauterine Growth Outcomes

Hormones are critical for appropriate fetal growth and development: they function in concert to necessitate organ development and are also involved in critical processes that differentiate the male from the female in utero. Although there is a clear understanding that specific hormones are required for the regulation of fetal growth and development, it is less clear whether these hormones drive sex-specific intrauterine growth outcomes. Despite these limitations, work from our groups and others have identified an important role for glucocorticoids and androgens in regulating changes in fetal growth and development.

### 4.1. Placental Glucocorticoid Signalling and Its Impact on Fetal Growth and Developmental Outcomes

Glucocorticoids, such as cortisol, bind to and exert their biological function through the glucocorticoid receptor (GR). This ligand-receptor interaction can result in the transcriptional regulation of up to 20% of the genes encoded in the human genome [52]. However, it is well established that glucocorticoids primarily function to exert anti-inflammatory effects [53]. Studies have also shown that glucocorticoids can suppress growth in several cell lineages including placental [54,55,56,57]. Disrupted glucocorticoid-mediated signalling can result in cellular dysfunction and is associated with the pathophysiology of several intrauterine growth perturbations, as well as intrauterine morbidity and mortality [58]. The placenta therefore has several mechanisms to protect the developing fetus from excessive exposure of glucocorticoid concentrations and potential dysregulation of this signalling axis [59,60,61].

Initial intrauterine glucocorticoid exposure may be altered by specific transmembrane transport proteins. One such protein, P-glycoprotein (P-gp, MDR1, or ABCB1) is involved in the export of intracellular cortisol [62], as well as a range of other endogenous or exogenous chemicals [63]. Although P-gp has been shown to be involved in placental glucocorticoid uptake and efflux that results in altered GR activity [59], there are limited studies that have reported sex-specific differences in its expression or activity. Using a mouse model of stress in mid-gestation, Wieczorek et al. [64] reported increased mRNA expression of *Abcb1a* in female placentae in response to stress. A similar response was observed in males, but did not reach statistical significance; however, the expression of *Abcc1* (involved in corticosteroid export) was significantly increased in males. These findings may suggest that the presence of maternal stress, and subsequent increase in maternal glucocorticoid concentrations, results in a more robust response in males to reduce in utero corticosteroid exposure. The relevance of these findings requires further investigation but may be a male-specific adaptation to mitigate potential anti-proliferative effects of glucocorticoids. Human population studies have demonstrated significantly reduced P-gp mRNA and protein expression in SGA placentae [65], further indicating dysfunction of this protein may have potential consequences for fetal growth outcomes.

Inactivation of cortisol to cortisone, via 11β-hydroxysteroid dehydrogenase type 2 (11β-HSD2), is also thought to be involved in reducing excess in utero glucocorticoid exposure. As such, dysfunction of this enzyme in the placenta may contribute to reduced intrauterine growth. Indeed, 11β-HSD2^−/−^ mice have reduced placental weight at 15d and 18d gestation, reduced fetal weight at 18d, increased placental *Slc38a2* and *Slc38a4* expression at 15d, but decreased *Slc2a3*, *Vegfa*, and peroxisome proliferator-activated receptor gamma (*Pparγ*) at 18d [66]; however, the effect of sex was not examined. In mice, maternal excess glucocorticoid exposure via osmotic minipump delivery has been shown to increase placental *Hsd11b2* expression in both sexes. Interestingly, once the delivery of glucocorticoids was stopped, placental *Hsd11b2* expression declined significantly in males (65% reduction relative to control males), whereas—in females—expression was comparable between groups [67]. Although functional activity was not determined, these findings would indicate sex-specific differences in placental *Hsd11b2* expression after excess in utero glucocorticoid exposure exist, which may increase male vulnerability in the presence of subsequent in utero insults associated with elevated glucocorticoid concentrations.

Some studies in human population cohorts have demonstrated sex-specific differences in the activity of placental 11β-HSD2. In appropriate for gestational age (AGA) term placentae, the activity of 11β-HSD2 was significantly reduced in females when compared with males [68]. The same study also reported increased 11β-HSD2 activity in female SGA placentae when compared with female AGA placentae, but this same response was not observed in males. In preterm placentae, 11β-HSD2 activity was significantly increased in females treated with betamethasone <72 h from the time of delivery and this was associated with an increase in umbilical arterial cortisol concentrations, when compared with males [69]. These observed sex differences in 11β-HSD2 activity in the presence and absence of pregnancy-specific complications may contribute to altered feto-placental glucocorticoid signalling.

There are limited studies that have examined how the absence of glucocorticoid signalling in utero impacts fetal growth and development. Cole et al. [70] first demonstrated that the absence of intrauterine glucocorticoid signalling resulted in poorly developed lungs and subsequent neonatal demise in a global GR knockout (GRKO) mouse model. More recent studies in global GRKO mice demonstrated significantly reduced heart size at 14.5–18.5d gestation [71]; however, the impact of GRKO on growth outcomes was not reported in either study. While no study to date has reported the impact of absent intrauterine glucocorticoid signalling on fetal growth outcomes, in vivo studies of excess prenatal glucocorticoid exposure and human studies of chronic maternal stress associated with elevated maternal glucocorticoid concentrations have identified how this hormone may modulate placental structure and function and subsequent fetal growth outcomes.

A rat model of dexamethasone-induced fetal and placental growth restriction identified significantly reduced labyrinth-zone expression of the *Vegf-A* isoforms *Vegf120* and *Vegf188*, as well as reducing the absolute volumes and surface areas associated with maternal and fetal blood spaces [72]. The study further reported that the absolute placental tissue volume was reduced by 36%, as were the size and relative density of fetal capillaries in the dexamethasone treated group, but the effect of sex was not addressed. However, some studies have identified that excess maternal glucocorticoid concentrations result in sex-specific alterations to placental function. A recent study of prenatal dexamethasone exposure in rats reported that the theoretical oxygen diffusion capacity was significantly reduced in placentae of males, relative to females [73]. Additionally, while *Igf1* expression was significantly reduced in both female and male dexamethasone-exposed placentae, *Igf2* expression was significantly upregulated in females only. In support of this, female placentae had upregulated glucose transporter (GLUT) 1, GLUT3, lipoprotein lipase (LPL), large neutral amino acid transporter (LAT1) and scavenger receptor class B, type 1 (SRB1) mRNA and protein expression, whereas in males the opposite was observed.

In mice, Cuffe et al. performed two separate studies [74,75] to investigate if exposure to synthetic or natural glucocorticoids induced similar sex-specific differences to the developing placenta. Similar to the findings of Hewitt et al. [72], dexamethasone exposure induced fetal growth restriction in both sexes, but reduced junctional zone area in females only [74]. This female-specific placental adaptation was associated with changes in the expression of certain GR isoforms and increased expression of apoptotic genes [75]. In contrast, corticosterone had no direct impact on fetal or placental growth but following removal of the glucocorticoid challenge male placentae had a significant increase in total placental weight that was thought to be due to an increase in junctional zone, but decrease in labyrinth zone, volume. The same study also demonstrated corticosterone increased *Nr3c1* expression, as well as the growth stimulating genes *Vegfa*, kinase insert domain receptor (*Kdr*), *Igf2* and mitogen-activated protein kinase kinase 1 (*Map2k1*) in male placentae only [67]. Intriguingly, glucocorticoids have been shown to inhibit the expression of some of these growth stimulating genes [72,76,77,78], which may suggest a level of glucocorticoid-resistance is initiated in the male placentae exposed to increased corticosterone concentrations. However, these findings, in part, contrast those reported by Guo et al. [73]. These discrepancies may be explained by the timing, severity, and duration of the in utero insult [79], species-specific differences in placental glucocorticoid-mediated signalling [80], or differential effects of synthetic and natural glucocorticoids on glucocorticoid-mediated signalling [81,82,83].

Changes in GR expression and localisation may contribute to sex-specific differences in growth outcomes. Placentae from IUGR females have been shown to increase nuclear GRα expression in both extravillous trophoblast (EVT) and syncytiotrophoblast cells (as measured by immunohistochemistry in humans) [84] and increased nuclear expression of GRαD1–3 isoforms (as measured by Western blot in mice) [75]. Unlike in females, growth restriction in males was associated with increased GRβ expression in humans [84], and a decrease in the activity of GrαA in mice, as determined by its cytoplasmic sequestration [75]. These sex-specific differences in the placental responsivity to changes in glucocorticoids favour the work conducted in human population studies of maternal asthma associated with sex-specific intrauterine growth outcomes. Saif et al. [60,85] demonstrated male placentae in the presence of maternal asthma significantly increased levels of the antagonistic isoform GRβ, and a low-transactivational isoform GRαD1, whereas female feto-placental units remain responsive to glucocorticoids due to increased GRαA expression and have reduced growth outcomes. Similar findings were reported in a sheep model of maternal allergic asthma, irrespective of sex [86]. Indeed, GRβ has been identified to confer glucocorticoid resistance in certain cell lines [87,88] by forming a non-responsive heterodimer with GRα, thereby inhibiting activity necessary for the transcriptional regulation of glucocorticoid-mediated genes. Structurally, GRβ lacks helix 12 in the ligand binding domain (LBD) needed for glucocorticoid binding [88,89] which further highlights the reduction of glucocorticoid responsivity in systems with increased GRβ levels. Overexpression of GRβ inhibits glucocorticoid-associated catabolism which was shown to increase cellular growth and proliferation associated with myogenesis [90]. Taken together, data from both and animal studies indicate female growth restriction may be induced by canonical glucocorticoid-mediated signalling. In contrast, the mechanisms contributing to disrupted male growth appear to be independent of glucocorticoid-mediated signalling and is likely driven by alternative pathways.

### 4.2. Placental Androgen Signalling: A Potential Regulator of Sex-Specific Intrauterine Growth and Developmental Outcomes

Despite convincing evidence of males having increased growth rates and birthweight outcomes, the mechanisms contributing to these sex-specific outcomes remain unclear. However, the anabolic actions of androgens may be a factor by which male-specific growth rates and outcomes are regulated. Given this, there is increasing interest in the function of androgen signalling in regulating feto-placental growth and development [91].

Throughout gestation, the levels of maternal circulating androgens including testosterone, dihydrotestosterone (DHT), dehydroepiandrosterone (DHEA), and androstenedione (A4) increase three-fold by the third trimester when compared to non-pregnant levels [92]. There are sex differences in the concentrations of circulating fetal and maternal androgens [93,94,95], with levels higher in male fetuses and in the maternal circulation of individuals carrying a male fetus. Fetal derived androgens are readily aromatised to estrogens by the placental cytochrome P450 (CYP) enzyme, CYP19A1 (aromatase). These fetal-derived androgens are required for placental-biosynthesised estrogens, an important physiological process that modulates uteroplacental vasculature [96,97,98,99]. Although the placenta is known to be involved in the aromatisation of androgens, other studies have reported placental-specific androgen biosynthesis. Escobar et al. 2011 [100] reported third trimester human placentae were able to synthesise androgens de novo. More recently, levels of tissue-specific androgens were measurable in first trimester human male placentae [95]; the levels of DHEA, A4, testosterone, and DHT were all detectable, with some of these androgens having higher levels when compared with liver-, adrenal-, or testis-specific levels. In line with these findings, the same study reported expression of genes involved in androgen biosynthesis in the male placenta. It is important to note that the study did not examine whether these changes in placental-derived androgens or steroidogenic genes varied between the sexes. Despite this, recent work identified that in male trophoblasts, DHT was an important upstream regulator of sex-specific differentially expressed transcripts [26]. This finding may be indicative of a male-specific preference for androgen-mediated signalling in the placenta. Indeed, expression levels of 5α-reductase, an enzyme involved in the reduction of testosterone into the non-aromatisable androgen, DHT, is increased in male placentae from uncomplicated pregnancies when compared with gestation-matched females [101]. Therefore, while the placenta may be critical for estrogen biosynthesis via androgen aromatisation, it also appears that this organ is involved in the biosynthesis of several androgens, which may be important in the regulation of sex-specific transcript expression. However, given certain androgens are undetectable within the fetal circulation [95], it is unclear whether placental-derived androgens function at an endocrine or paracrine signalling level to necessitate transactivation of target genes.

Although evidence of sex-specific differences in intrauterine growth outcomes exists, there are limited studies that have examined to what extent androgens influence this. Disrupted androgen signalling via various mutations in the AR gene results in 46,XY karyotyped neonates presenting with female-like phenotypes [102,103]. These 46,XY neonates were reported to have significantly reduced birthweight adjusted for gestational age when compared to a control male population [103]. Similar findings have been reported in a global androgen receptor knockout (ARKO) mouse model [104]. It appears androgens and their receptors are critical for appropriate male growth and development in utero and any perturbation to this signalling pathway can result in adverse outcomes.

Human population studies have demonstrated SGA male neonates have significantly reduced levels of testosterone when compared to appropriate for gestational age (AGA) neonates [105]. Similarly, Carlsen et al. [106] demonstrated negative associations between testosterone concentrations at 17 and 33 weeks’ gestation and birthweight in female neonates. In contrast, Voegtline et al. [107] reported reduced male birthweight, but increased postnatal weight gain in pregnancies with high compared to low maternal plasma testosterone concentrations. The same study reported increased female birthweight in the high testosterone group compared to the low testosterone group, but no change in postnatal weight gain. These discrepancies between studies are likely products of study design (i.e., populations used), which highlights the need for additional research investigating associations between androgen concentrations and birthweight outcomes, especially when considering a specific pregnancy comorbidity.

Sex differences in fetal growth in the presence of specific pregnancy complications may be due to altered androgen concentrations. Previously, sex-specific birthweight outcomes from pregnancies complicated by mild PE have been reported [19]. Specifically, female neonates from preeclamptic pregnancies have a significantly reduced birthweight centile (BWC) when compared to neonates from healthy control groups. In contrast, male BWC remained unaltered between normotensive and PE groups. Given previous studies have shown increased testosterone concentrations in pregnancies complicated by PE and a male fetus [108,109], it is possible that androgens have a role in regulating the differential intrauterine growth response between the sexes. This is supported, in part, by the identification of sex-specific dysregulation of androgen biosynthesis from placentae of PE pregnancies, which demonstrated decreased CYP19A1 mRNA and protein expression in males, relative to females [110]. Interestingly, female placentae of PE pregnancies had increased expression of CYP19A1 compared to female placentae from an uncomplicated pregnancy. Although the activity of aromatase was not measured, these findings, in conjunction with a reported increase in androgen concentrations in patients with PE and a male fetus suggest that in the presence of a pregnancy complication, males may prioritise androgen bioavailability by suppressing aromatase expression. Likewise, a study conducted by Maliqueo et al. [111] reported reduced aromatase activity in placentae from patients diagnosed with polycystic ovarian syndrome (PCOS), when compared to controls, irrespective of sex. The same study also reported lower A4 and higher estriol concentrations in the cord blood of female newborns of patients with PCOS, further supporting a female-specific preference for eliminating excess circulating androgens, an adaptation which may reduce the risk of fetal virilisation [112,113,114] and the development of certain diseases, including PCOS later in life [115,116,117].

Animal studies have demonstrated that high levels of androgens during gestation can lead to IUGR-like outcomes, reduced fetal viability, and reduced litter size [118,119,120,121,122]. Cleys et al. [119] reported that the average birthweight of female offspring of testosterone propionate (TP) treated ewe was significantly reduced compared to controls, and that no difference in male birthweights were observed between treatment groups. These findings are in agreement with previous research conducted by Beckett et al. [118], which identified consistently lower birthweights of female offspring born to aromatisable (testosterone) and non-aromatisable (DHT) androgen treated ewes. In contrast, Sathishkumar et al. [122] reported in a rodent model that increased maternal testosterone concentrations suppressed fetal growth indirectly by affecting placental amino acid delivery to the fetus. Prenatal TP exposure significantly reduced SLC38A4 mRNA and protein expression in both male and female rat placentae and was associated with reduced placental amino acid transport capacity with a greater effect observed in males. Further prenatal TP studies by the same group reported reduced uterine blood flow, reduced spiral artery elongation, and reduced placental oxygenation, which in turn resulted in increased fetal and placental hypoxia [121]. The same study identified several genes involved in vasculogenesis and angiogenesis to be sex-specifically altered in placentae of TP exposed dams. Specifically, nitric oxide synthase (*Nos3*)*,* angiopoietin-like 4 (*Angptl4*)*,* endomucin (*Emcn*)*,* endothelin 1 (*Edn1*)*, Flt1*, and *sFlt* were upregulated in male placentae of TP exposed dams, whereas C-X-X chemokine receptor type 4 (*Cxcr4*)*,* bone morphogenic protein 4 (*Bmp4*)*,* urokinase (*Plau*) and interleukin (IL) 1 beta (*IL1β*) were upregulated in female placentae of TP exposed dams. In line with these molecular differences, sex-specific functional differences were observed. Male placentae of TP exposed dams had a greater reduction in oxygen delivery, as determined by pimonidazole binding, and were more hypoxic when compared with females.

Additional sex-specific alterations in gene expression profiles have been observed in a study conducted by Kelly et al. [123]. Using a prenatal TP sheep model of PCOS, an increase in the mRNA expression of *IL-1β*, C-C motif chemokine ligand (*CCL2*), *VEGF*, and *HIF1α*, and a decrease in the mRNA expression of *IL-6* was observed in female placentae of TP treated ewe. In contrast, only *IL-1β* and *CCL2* mRNA expression were decreased in male placentae of TP treated ewe, when compared with control. Intriguingly, research in prostate cancer cells demonstrated *CCL2* is repressed by activated androgen signalling [124]. Therefore, this sexual-dimorphism in *CCL2* expression in sheep placentae in response to an excess of androgens suggests a male-specific bias to preferentially maintain androgen-dependent signalling throughout gestation, whereas female placental androgen signalling may be suppressed. This observed sexual dimorphism further indicates a priority for females to enhance placental reserve capacity, independent of the insult by which oxygen and nutrient supply is reduced, whereas males invest in mechanisms that necessitate growth outcomes.

Androgen signalling primarily occurs through the androgen receptor (AR), a steroid receptor belonging to the nuclear receptor 3-ketosteroid group C (NR3C) subfamily. The *AR* gene has been localised to Xq11-12, contains eight exons and codes for a 919-amino acid protein. Alternative splicing of AR pre-mRNA can result in at least 20 transcript variants [125,126,127,128,129,130,131] that gives rise to numerous protein variants with molecular weights (MW) ranging from 45–120 kDa. Expression of total AR was initially reported in the first trimester human placenta, where its expression was localised to the cytoplasm and nucleus of decidual and trophoblast cells [132]. Since then, numerous studies have identified placental expression of AR at the mRNA and protein level [50,110,118,119,133,134,135]. Recently, it was reported that the human placenta expressed multiple AR protein isoforms that vary in relation to feto-placental sex and the presence and absence of the complication of maternal asthma [50]. The findings reported that in males, the expression of the functional full length AR isoform, AR-FL, was significantly reduced in the presence of maternal asthma whereas the expression of the N-terminally truncated isoform, AR-45, was significantly increased and positively associated with male neonatal growth outcomes and androgen-mediated downstream growth target genes. This novel finding of multiple placental AR isoforms was recently supported in sheep placenta, independent of sex [134]. It has been proposed that the preferential expression of AR protein isoforms in male placentae in the presence of specific pregnancy complications contributes to maintained intrauterine growth; however, in the presence of multiple maternal complications this adaptation is compromised, thereby contributing to disrupted placental androgen signalling and reduced intrauterine growth for the male fetus [50,91]. Indeed, the identification and continued characterisation of placental AR protein isoforms further challenges our current understanding of placental androgen signalling and highlights the complexity of an already poorly understood signalling axis, but has provided greater insight into mechanisms contributing to sex-specific intrauterine outcomes in the presence and absence of an adverse maternal environment.

## 5. Conclusions

**Figure 3:**
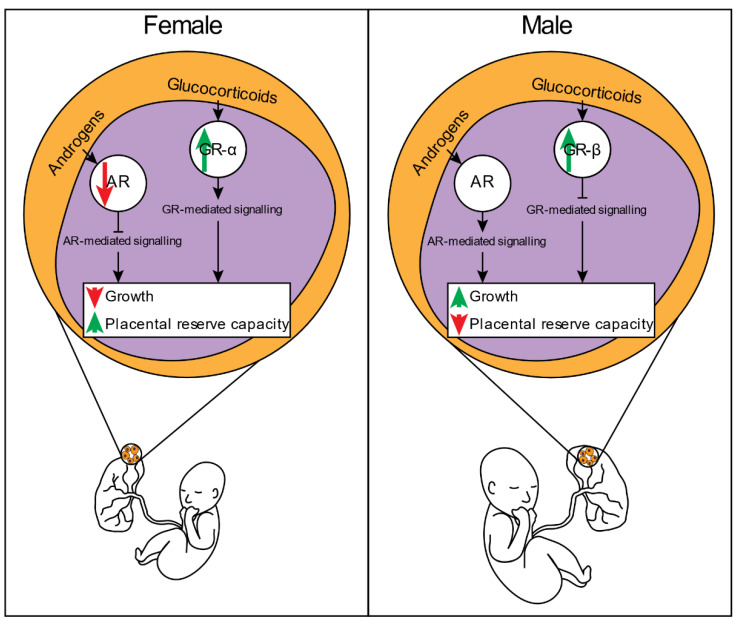
Sex-specific differences in androgen and glucocorticoid-mediated signalling within the feto-placental unit. Females prioritise pathways regulated by glucocorticoids to enhance placental reserve capacity at the detriment of growth, whereas males prioritise androgen-mediated signalling pathways to enhance growth at the expense of placental reserve capacity.

The underlying mechanisms that drive sex-specific in utero growth differences and the consequential outcomes are mediated through both placental and non-placental pathways. The current literature suggests a male-specific preference for growth signalling pathways throughout gestation, whereas females appear to prioritise pathways that increase feto-placental adaptability and placental reserve capacity. In the presence of a maternal complication, the increased adaptability of a female feto-placental unit may reduce intrauterine morbidity and mortality rates when compared to males exposed to a comparable in utero environment. The mechanisms by which these sex differences in feto-placental function are regulated may, in part, be due to alterations in the unit’s responsivity to specific hormones: in general, females appear to increase glucocorticoid sensitivity, whereas males favour signalling pathways mediated by androgens (Figure 3). However, more studies are needed to untangle the complexity of the feto-placental hormonal milieu in regulating intrauterine growth and developmental outcomes and how steroid signalling axes may be disrupted in the presence of pregnancy complications. By continuing to expand our knowledge of mechanisms that mediate sex-specific in utero growth and developmental outcomes, future studies will be equipped with a greater repertoire of pathways to identify and potentially target. These advancements will undoubtedly reduce the prevalence of perinatal morbidities and mortality and could also provide opportunity to explore sex-specific therapies and interventions for the at-risk fetus, given the observed dimorphic nature of pathways preferentially regulated between the sexes.
